# The effect of Aβ seeding is dependent on the presence of knock-in genes in the *App*^*NL*−*G*−*F*^ mice

**DOI:** 10.3389/frdem.2022.941879

**Published:** 2022-09-12

**Authors:** Sean G. Lacoursiere, Jiri Safar, David Westaway, Majid H. Mohajerani, Robert J. Sutherland

**Affiliations:** ^1^Canadian Centre for Behavioural Neuroscience, University of Lethbridge, Lethbridge, AB, Canada; ^2^Departments of Pathology, Neurology, Psychiatry, and National Prion Disease Pathology Surveillance Center, School of Medicine, Case Western Reserve University, Cleveland, OH, United States; ^3^Centre for Prions and Protein Folding Diseases, University of Alberta, Edmonton, AB, Canada

**Keywords:** seeding, Alzheimer's disease, microglia, mice, memory, amyloid-β

## Abstract

Alzheimer's disease (AD) is characterized by the prion-like propagation of amyloid-β (Aβ). However, the role of Aβ in cognitive impairment is still unclear. To determine the causal role of Aβ in AD, we intracerebrally seeded the entorhinal cortex of a 2-month-old *App*^*NL*−*G*−*F*^ mouse model with an Aβ peptide derived from patients who died from rapidly progressing AD. When the mice were 3 months of age or 1 month following seeding, spatial learning and memory were tested using the Morris water task. Immunohistochemical labeling showed seeding with the Aβ was found accelerate Aβ plaque deposition and microgliosis in the *App*^*NL*−*G*−*F*^ mice, but this was dependent on the presence of the knocked-in genes. However, we found no correlation between pathology and spatial performance. The results of the present study show the seeding effects in the *App*^*NL*−*G*−*F*^ knock-in model, and how these are dependent on the presence of a humanized *App* gene. But these pathological changes were not initially causal in memory impairment.

## Introduction

Alzheimer's disease (AD) is the most common form of dementia, affects millions of people, and has a high social and monetary cost (Alzheimer's Association, 2019). It is characterized by stereotypical pathological stages of amyloid-β (Aβ) aggregation and neurofibrillary tangle formation that are progressive (Braak and Braak, 1991; Ettcheto et al., 2018)—this protein aggregation is assumed to be central to AD pathogenesis (Friesen and Meyer-Luehmann, 2019; McAllister et al., 2020). Progression of pathology is associated with memory loss, impaired thinking skills, and, eventually, impairments in all facets of life (Matteson et al., 1996; Ferri et al., 2004; de Vugt et al., 2005). The cause of AD may be a prion-like spread of Aβ, resulting in neuroinflammation, plaque deposition, and hyperphosphorylation of tau, ultimately causing synapse loss and brain atrophy (Harper and Lansbury, 1997; Bloom, 2014; Walker et al., 2018).

Intracerebral seeding has become an effective tool to understand the role of protein aggregation in neurodegenerative diseases as it allows the control over the spatial and temporal onset of amyloidosis (Friesen and Meyer-Luehmann, 2019) as seeding Aβ accelerates the deposition of Aβ plaque *in vivo* in a prion-like manner (Walker et al., 2016; Olsson et al., 2018), in which native Aβ species are misfolded following the template and conformational properties of the seeded Aβ (Come et al., 1993; Eisele, 2013). The effects of seeding are also dependent on the genotype of the host: Mice without mutations in *App* or only possessing murine *App* do not show this effect, or, if they do, the required incubation time increases significantly before effects are seen (Meyer-Leuhmann et al., 2006; Eisele et al., 2009; Friesen and Meyer-Luehmann, 2019).

Much of the intracerebral seeding work has been done using first-generation mouse models using synthetic or murine Aβ (McAllister et al., 2020); however, due to the presence of APP artifacts in the first-generation mouse models, the conclusions drawn about the correlation between Aβ pathology, the effects of seeding, and the behavioral outcomes are in question (Sasaguri et al., 2017, 2022). Recently, the single knock-in *App*^*NL*−*G*−*F*^ mouse model has been developed (Saito et al., 2014). To develop this second-generation, knock-in model, the murine Aβ sequence was first humanized, and Swedish, Beyreuther/Iberian, and Arctic mutations were inserted. The Swedish (NL) mutation (KM670/671NL) increases the production of APPβ and the C-terminal fragment containing the entire Aβ sequence in neuronal cells (Shin et al., 2010). The Beyreuther/Iberian (F) mutation (I716F) increases the ratio of Aβ_42_ to Aβ_40_ and APP C-terminal fragments but also decreases the APP intracellular domain production; this is thought to be due to a reduction in APP proteolysis by γ-secretase due to the mutation leading to a protein that is poorly processed by γ-secretase (Guardia-Laguarta et al., 2010). The Arctic mutation (G) alters binding properties of various antibodies to Aβ for immunohistochemistry (Saito et al., 2014).

Several studies have characterized the development of pathology, functional connectivity, and behavioral phenotypes of these mice under multiple conditions (Jafari et al., 2018; Latif-Hernandez et al., 2019, 2020; Mehla et al., 2019; Upite et al., 2020). Of the studies using this for Aβ seeding, both studies found seeding accelerated Aβ deposition, but neither tested behavior of the mice following the seeding (Purro et al., 2018; Ruiz-Riquelme et al., 2018). To address the paucity of behavioral testing immediately following seeding, we seeded human Aβ into the *App*^*NL*−*G*−*F*^ mouse model.

Here, we wanted to determine whether seeding Aβ would cause immediate impairment in spatial learning and memory in the single *App* knock-in mouse model and to further understand how the presence of mutations influenced the effects of seeding on Aβ deposition but also on the response of microglia. We used young mice to determine if the initial Aβ deposition caused cognitive impairment without any other endogenously generated pathology and cognitive impairment.

First, we predicted that the mice seeded with Aβ would show a reduction in spatial learning and memory; second, it is predicted that the presence and absence of the knocked-in mutations would determine the seeding effects and the level of cognitive impairment. Finally, we predicted that the Aβ seeding would increase activated microglia. Overall, we found that the effects of seeding human HPC tissue and Aβ containing tissue were dependent on the presence of the NL G F knock-in genes.

## Methods

### Subjects

Fifty-three single knock-in *App* mice were seeded in this study. Similar number of male and female mice was used (31 men, 22 women) as no difference between sexes was found in previous characterization (Mehla et al., 2019). The mice were caged in standard housing, 2–5 mice per cage, and kept on a 12-h light/dark cycle. The mice were given *ad libitum* food and water. The mice were handled prior to behavioral testing, which was completed at approximately the same time during the light cycle by an experimenter blinded to the conditions.

The mice were created by crossing mice from the RIKEN institute and *C57Bl/6J* mice. The mice were grouped based on genotype and seed. The mice were either homozygous negative (*App*^−/−^), carrying no mutations; heterozygous (*App*^+/−^), carrying only one copy of the Swedish, Beyreuther/Iberian, and Arctic mutations; and homozygous positive (*App*^+/+^), carrying two copies of the knocked-in mutations. The mice were then randomly assigned to be seeded with the control or rpAD seed. See Table 1 for final grouping.

**Table 1:** Grouping of genotype and seed used for behavioral testing (and immunohistochemical analysis).

**Genotype**	**Control**	**rpAD**
*App^−/−^*	15 (3)	15 (3)
*App^+/−^*	7 (3)	3 (3)
*App^+/+^*	4 (2)	9 (3)

### Genotyping

Punched mouse ear tissue was subjected to DNA extraction and PCR cycling using a Millipore-Sigma's RedExtract-N-Amp Tissue PCR kit (XNAT-100RXN). PCR cycler condition: 94°C for 3 min, 94°C for 30 s, 57°C for 45 s, 72°C for 1 min x 35 cycles. Stored at 4°C. Primer sequences were obtained from the Riken Institute: E16WT: 5′-ATC TCggAAgTgAAgATg-3′; E16MT: 5′-ATCTCggAAgTgAAT CTA-3′; WT: 5′-TgTAgATgAgAA CTT AAC-3′; loxP: 5′-CgT ATA ATgTATgCT ATA CgA Ag-3′. PCR products were loaded onto agarose gel electrophoresis for visualization, with a wild-type band at 700 bp and a mutant band at 400 bp.

### Aβ seed

The Aβ seeds were obtained from human hippocampal tissue (The National Prion Disease Pathology Surveillance Center at Case Western Reserve University Medical School), assessed for purity, and stereotaxically injected. The University Hospitals Institutional Review Board (IRB) approval is for all autopsied (“discarded”) human tissues, and all samples are anonymized (coded) and handled in compliance with the NIH policy to protect privacy. The type of seed was determined by the rate of AD progression. The biochemical analysis for the control tissue showed effectively zero Aβ (D/N) ratio Aβ_40_ or Aβ_42_, but Aβ%, according to sedimentation velocity in a calibrated sucrose ingredient, showed between ~8 and 12% Aβ_40_ and Aβ_42_ for the controls (Cohen et al., 2015). Furthermore, the rpAD seed showed higher levels of Aβ_42_ particles between 30 and 100 monomers with few particles <30 monomers; therefore, the rpAD seed particles are of higher molecular weight. It is these monomers that trigger the initial phase of Aβ seeding (Katzmarski et al., 2019). All brain tissue homogenate was buffered with phosphate-buffered saline (PBS) at a pH of 7.4 and kept at −80°C. The seed was 10% w/v. Prior to possession of the tissue, the tissue underwent several selection criteria steps.

1. Referral to the National Prion Disease Pathology Surveillance Center to classify any prion disease.
2. Six or more MMSE points of decline per year.
3. Absence of autosomal dominant AD patterns.
4. Absence of mutations in human prion protein.
5. Aβ and tau proteins resembling sporadic Alzheimer's disease.
6. No other neuropathological comorbidity.
7. All results within 85% confidence interval.

Another five inclusion criteria for the classical Alzheimer's disease tissue were used, they are as follows:

1. Clear clinical diagnosis of Alzheimer's disease.
2. No autosomal dominant patterns of dementia.
3. Alzheimer's disease based on tau and Aβ proteins.
4. No comorbidity with other neuropathological diseases.
5. Results within 95% confidence interval.

### Stereotaxic intracerebral seeding surgery

The mice were subcutaneously injected with buprenorphine (Vetergesic; 0.05 mg/kg; concentration = 0.03 mg/ml) 30 min prior to anesthesia induction (isoflurane). The oxygen flow rate for induction was between 4 and 5 L/min, and isoflurane was increased in a stepwise manner to a maximum of 5 L/min. Oxygen and anesthesia flow rates were reduced to 0.9 and 1.5–3 L/min, respectively, for the duration of the surgery. After the head was shaved, the scalp was cleaned with 4% stanhexidine (Omegalab), followed by 70% isopropyl alcohol. Lidocaine (0.1 ml of 0.2%; Rafter8) was subcutaneously injected under the scalp. Bregma was used to find the stereotaxic coordinates for the medial entorhinal cortex (Allen Institute for Brain Science, 2016). The coordinates used for injection were AP: −4.48, ML: 3.00, DV: 3.44 to target the medial entorhinal cortex. A 0.5-mm diameter hole was drilled through the skull to the brain at the coordinates.

The tissue homogenate was vortexed for 30 s before being loaded into the micropipette. Each mouse received 2 μL (1 μL/hemisphere) of the Aβ or control tissue homogenates. Seeding was performed with a Nanoject II (Drummond Scientific Company, PA) set to slowly inject 50.6 nL. Prior to seeding, a test injection was done to ensure proper flow, and the micropipette was cleaned with 70% isopropyl alcohol. The micropipette was inserted into the brain at the locations described and allowed to rest for 2 min before the first injection, with all following injections 20 s apart for a total of 20 injections. The micropipette stayed in place for 2 min after the final injection before being removed. A test injection was done again once the micropipette was removed, and the micropipette was cleaned with 70% isopropyl alcohol before the next hemisphere injection. The mice were kept on the same 12-h-light/dark cycle throughout recovery.

### Perfusions and sectioning

Following the behavioral testing, the mice were overdosed with an intraperitoneal injection of sodium pentobarbital and transcardially perfused with 1X phosphate-buffered saline (PBS) and 4% paraformaldehyde (PFA). The brains were fixed for 24 h in 4% PFA before being transferred to a solution of 30% sucrose solution for at least 3 days. The brains were sectioned on a frozen-sliding microtome at 40 μm in a 1:6 series and stored in 1X PBS + 0.02% sodium azide solution until staining. Prior to staining, the brain sections were mounted on super frost positively charged slides, allowed to dry up right for 30 min, and stored overnight at 4°C.

### Aβ deposition and microglia immunohistochemistry

The slides were rinsed in 4% PFA for 4 min, washed with 1X Tris Buffer Saline (TBS), and underwent a 70% Formic Acid wash. The slides were rinsed in 1X TBS, TBS-A (1X TBS + 0.1% Triton X), and TBS-B [TBS + 0.1% Triton X + 2% bovine serum albumin (BSA)]. The primaries used were: Anti-82E1 [an Anti-β-amyloid (N), IBL, 10323, mouse] 1:1,000 and Anti-Iba1 (Rabbit, 019-19741, Wako) −20C 1:1,000, 1 ml/slide in TBS-B for 2 days at RT in a dark humid chamber, sealed in a plastic wrap on the rotator at 50RPM. Following primary incubation, TBS, TBS-A, and TBS-B washes were repeated. Secondaries used: Anti-mouse-alexa-488 [IgG (H + L) goat, Abcam, ab150113] 1:1,000 and anti-rabbit-alexa-594 [IgG (H + L) goat, Invitrogen, A11037] 1:1,000 (1 ml/slide) in TBS-B overnight in a dark humid chamber sealed in a plastic wrap on the rotator at 50RPM. Secondary was washed with 1X TBS and cover slipped with Vectashield + DAPI.

### Imaging

Full-slide imaging was completed on a digital slide scanner (NanoZoomer 2.0-RS, HAMAMATSU, JAPAN) at 20X magnification. Slide images were exported using NDP.View 2. Quantification of Aβ plaques and microgliosis was done by pixel and object classification using iLastik (version 1.3.0-OSX) (Berg et al., 2019) and ImageJ (version 1.51 s). ILastik was trained to segment both plaque and activated microglia separately. Threshold values were between 0.3 and 0.4, with a size filter minimum of 10 pixels. Sections were processed from ~AP 1.7 to −4.77, and the count for each section was averaged within groups.

### Apparatus

The MWT pool was 1.55 m in diameter with water temperature maintained at 21 ± 1°C with a white, 12.5-cm submerged target platform and unobstructed distal cues surrounding the edges. A camera was fixed to the ceiling connected to a laptop with HVS Image 2100, which was used to track the swim patterns of the mice.

### Procedure

At 2 months of age, the mice were intracerebrally injected with a human tissue or the rpAD seed. One month following the seeding, the mice were tested using the MWT to determine if this seeding resulted in an impairment in spatial learning and memory. The mice were given four 30-s trials each day for 6 days. Starting position for each day was pseudo-randomized based on the cardinal starting locations, with each sequence of starting locations being different each day. On Day 7, the mice were tested using a no-platform probe. The temperature of the pool is kept at a cool 21°C to incentivize the mice to escape. Following the testing, tissue was collected to assess the extent of Aβ deposition and microgliosis.

### Statistics

Four parameters were measured during MWT training: the proximity of the mouse to the hidden platform, the time to find the platform, the path length, and the swim speed. Following the training, the mice were tested on a no-platform probe trial, and the amount of time spent in the target quadrant was compared to the opposing quadrant, and to the average of the two adjacent quadrants. For the pathology analysis, the number and the size of Aβ plaque throughout the brain were measured along with the number of microglia cells.

A two-way repeated measures ANOVA with a Dunnett's multiple comparison test was used to determine whether the mice significantly reduced their swim parameters from the 1st to the last day of the training and to determine whether the time spent in the target quadrant was influenced by the seed and the genotype, using the time in the target quadrant as the dependent variable. Probe test performance was also compared to chance performance (25%) using a single-tailed-paired sample *t*-test as we only wanted to know if performance was better than chance.

As the effects of seeding the rpAD seed were unknown, it is difficult to specify the effect size of interest. Furthermore, we were testing whether the seeding would have an effect or not, and not necessarily the size of the effect. Therefore, the resource equation method was used to determine if the sample size was sufficient. The error degrees of freedom were determined to be 47: six treatment groups subtracted from the total number of experimental units, 53 putting the error degrees of freedom far above the necessary amount to detect a specified effect (Festing et al., 2002).

All statistics were done using Prism 9 (mac OS).

## Results

**Figure 1:**
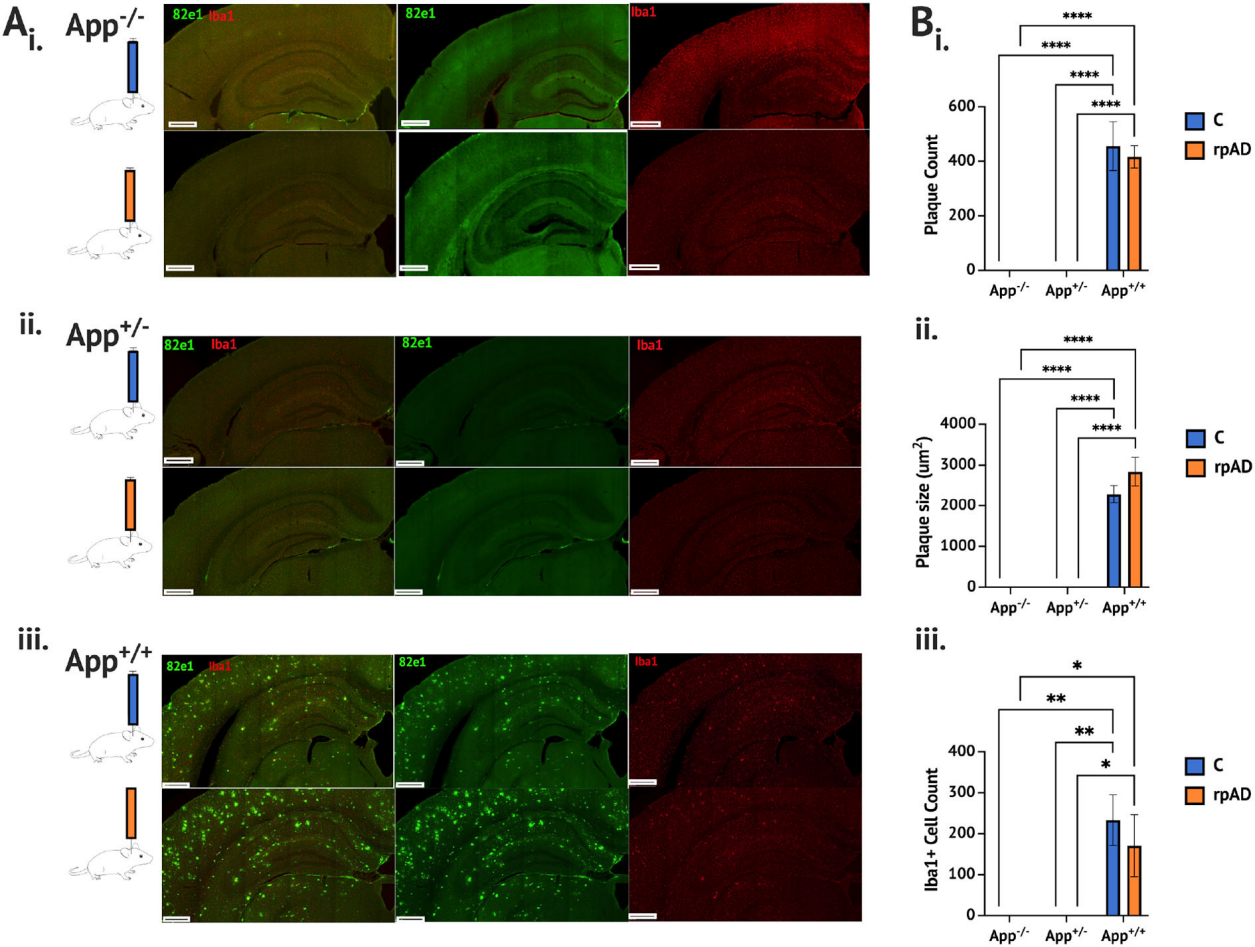
Aβ and microglia pathology analysis 1 month following seeding. **(A)** Photomicrographs of representative immunohistochemical staining of Aβ plaque (82e1, green) and microglia (Iba1, red), following seeding of the control HPC tissue (blue) and rpAD seed (orange) **(B)**. Quantification of plaque and microglia showing (i) the plaque count (ii) the plaque size, and (iii) the microglia was significantly increased in the *App*^+/+^ mice following seeding with the C and rpAD seed. Scale bar = 1 mm. *p < 0.05; **p < 0.01, ***p < 0.001; ****p < 0.0001.

Seeding either the control or rpAD seed into the *App*^+/+^ mice resulted in significant increase of a plaque count [*F*_(2, 11)_ = 136.1, *p* < 0.0001], plaque size [*F*_(2,11)_ = 150.4, *p* < 0.0001] and activated microglia cells [*F*_(2, 11)_ = 18.19, *p* = 0.0003] compared to both the *App*^−/−^ and *App*^+/−^; both of which showed no Aβ plaque pathology or activated microglia (Figure 1A and B). The seed was found to have no significant effect on the plaque count [*F*_(1, 11)_ = 0.303, *p* = 0.593], plaque size [*F*_(1, 11)_ = 1.64, *p* = 0.227], or activated microglia [*F*_(1, 11)_ = 0.472, *p* = 0.507]. See Supplementary Figure 1 for pathology assessment 4 months following the seeding.

**Figure 2:**
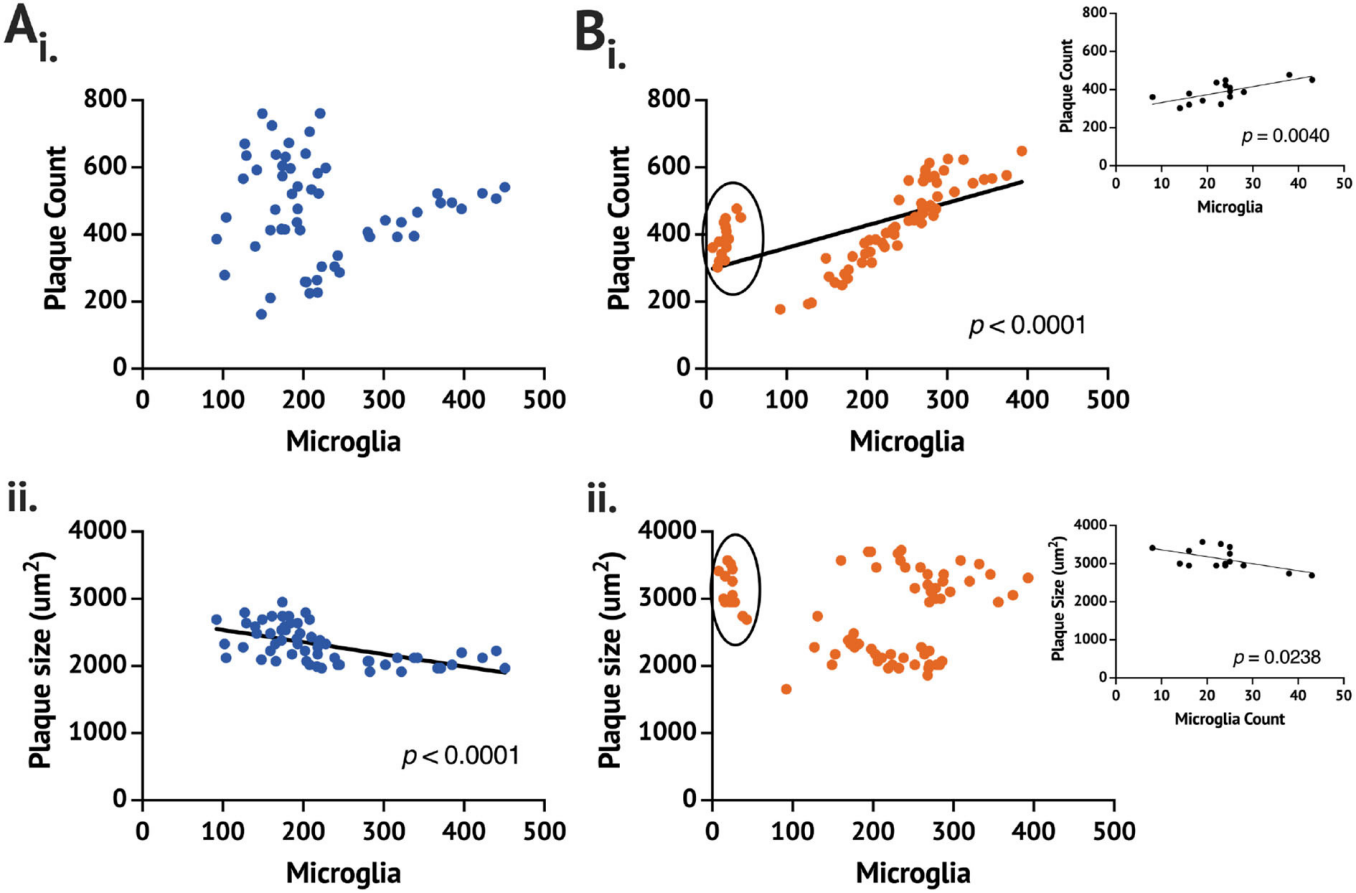
Correlation of the plaque and the microglia count 1 month following seeding in the *App*^+/+^ mice seeded with the **(A)** control (*n* = 2 brains,) and **(B)** rpAD seed (*n* = 3). Correlation is between the mean plaque count (i) or the plaque size (ii) and the number of counted microglia on full brain sections sampled from 1:6 sectioning series. Circled data in (Bi) and (Bii) are shown in the inset.

While no difference in the plaque count or size was found between the *App*^+/+^C and rpAD-seeded mice, the rpAD-seeded mice showed a significant correlation between the number of microglia and the number of plaques, whereas the *App*^+/+^ C mice did not show a significant correlation (Figure 2). A significant negative correlation was found for the number of activated microglia and the size of the plaque in the *App*^+/+^ C mice, but this correlation was not found in the rpAD mice.

From the data, it is clear that one App^+/+^ rpAD mouse had a comparable Aβ plaque count and size but no more than 50 activated microglia counted. With removing the low-microglia data and analyzing them separately, the plaque count and microglia remain a significantly positive correlation; however, the plaque size and the microglia count become a significant positive correlation (*p* = 0.0047). The individual App^+/+^ rpAD mouse with low microglia when analyzed separately shows a significantly positive correlation between the plaque count and microglia (*p* = 0.004), but the correlation between the plaque size and the microglia count is found to be a significantly negative correlation (*p* = 0.0238), where the plaque count and microglia count increased together, but, as microglia increased, there was a reduction in the plaque size.

**Figure 3:**
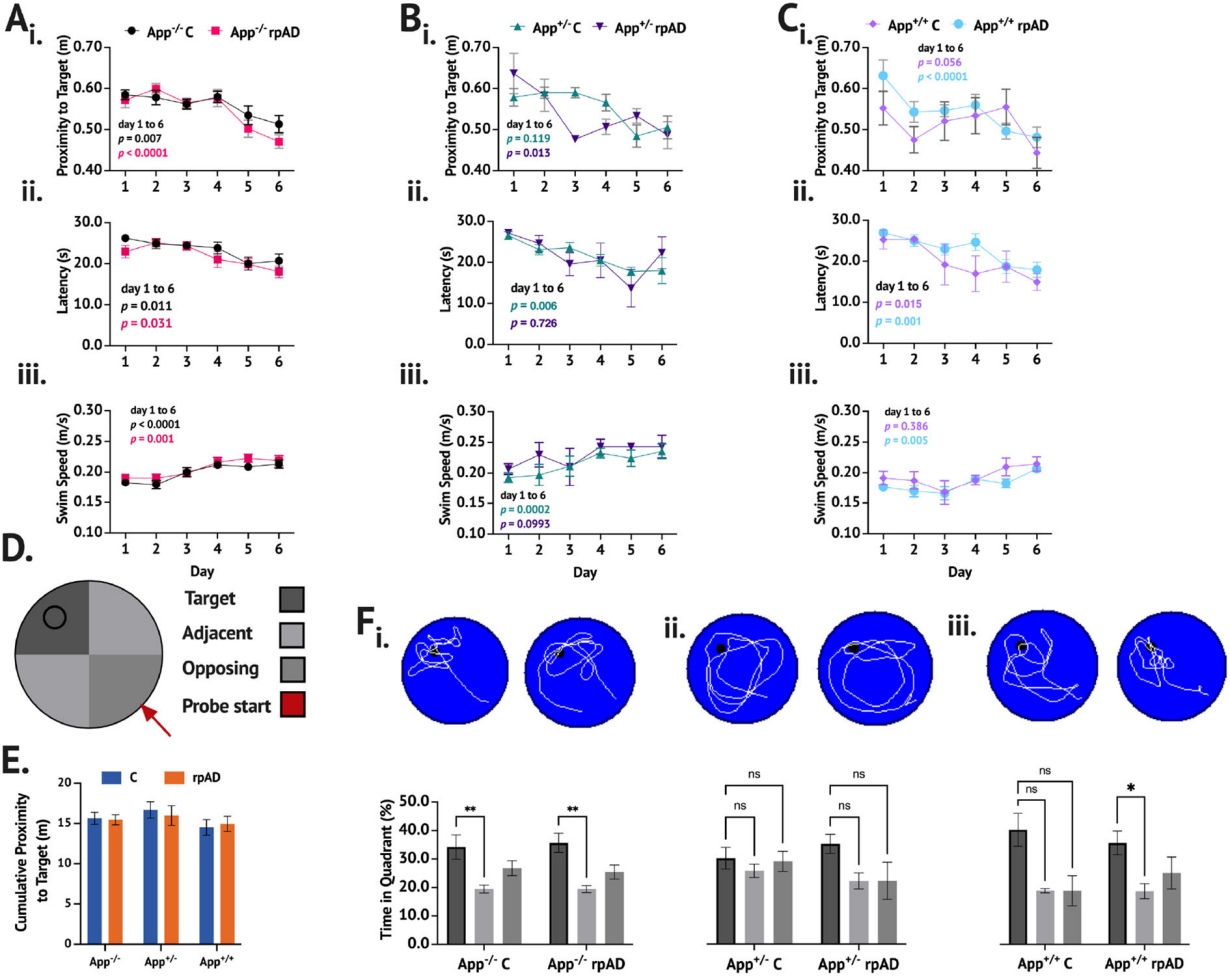
MWT performance following seeding. **(A–C)** show the performance of App^−/−^, App^+/−^, and App^+/+^ mice, respectively. (i.) Proximity to target, (ii.), latency to escape, and (iii.) swim speed were measured across six days of training. D. Representative schematic of pool set up. E. Cumulative proximity to target during probe trial showed no significant difference between groups. F. Representative swim path with a breakdown of time in quadrant immediately below for (i.) App^−/−^, (ii.) App^+/−^, and (iii.) App^+/+^ mice.

When trained and tested on the MWT (Figures 3A–F), the mice showed a significant reduction in proximity across training [*F*_(5, 235)_ = 14.41, *p* < 0.0001]. While no group differences were found [*F*_(5, 47)_ = 0.901, *p* = 0.489], a significant group × day interaction was found [*F*_(25, 235)_ = 1.690, *p* = 0.025]. The *App*^−/−^ C and *App*^−/−^ rpAD mice showed a significant reduction in proximity between the 1st and last days of the training (*p* < 0.01) and so did the *App*^+/−^ rpAD (*p* < 0.05) and *App*^+/+^ rpAD mice (*p* < 0.0001)—the *App*^+/+^ rpAD-seeded mice showed a significant reduction in proximity after 1 day of the training (*p* = 0.01). The *App*^+/−^ C mice did not show a significant reduction between the 1st and last days but did show a significant reduction by the 5th day; the *App*^+/+^ C mice did not show any significant reduction (Figure 3Ai).

The mice also showed a significant reduction in latency to escape across training [*F*_(5, 235)_ = 15.19, *p* < 0.0001], but no group differences [*F*_(5, 47)_ = 1.190, *p* = 0.329] or group x day interaction [*F*_(25, 235)_ = 0.969, *p* = 0.510] was found. All groups of the mice showed significant reduction in latency to escape. The *App*^−/−^C, *App*^−/−^ rpAD, *App*^+/−^ C, *App*^+/+^ C, and *App*^+/+^ rpAD all showed significant reduction in latency to escape by the final day (*p* < 0.05), but the *App*^+/−^ rpAD only showed significant reduction by the 5th day (Figure 3Aii).

The swim speed was found to increase significantly over the training [*F*_(5, 235)_ = 20.32, *p* < 0.0001], and significant differences between the groups were found [*F*_(5, 47)_ = 3.84, *p* = 0.005]; no group x day interaction was found [*F* (25, 235) = 1.23, *p* = 0.218]. All the groups, except for the App^+/−^ rpAD and *App*^+/+^ C significantly increased their swim from the 1st to last day1 of the training (*p* < 0.05). The *App*^+/−^ C and rpAD mice were both found to have an overall significantly faster swim speed than the *App*^+/+^ rpAD mice (*p* < 0.05; Figure 3Aiii).

In the no-platform probe trial within the genotype, comparisons were used to determine if the mice spent significantly more time in the target quadrant compared to the average of the two adjacent quadrants and the opposing quadrant (the opposing quadrant was the starting quadrant). Both the *App*^−/−^ C and rpAD mice spent significantly more time in the target quadrant compared to the non-target quadrants [*F*_(2, 56)_ = 10.83, *p* = 0.0001], but the seed had no effect [*F*_(1, 28)_ = 0.0003, *p* = 0.985]. Neither the *App*^+/−^ C or rpAD mice spent significantly more time in the target quadrant [*F*_(2, 16)_ = 1.98 *p* = 0.170], but, again, the seed had no significant effect on performance [*F*_(1,8)_ = 0.556, *p* = 0.477]. Within the *App*^+/+^ mice, a significant preference for the target was found [*F*_(2, 22)_ = 5.59, *p* = 0.012], with an overall preference for the target quadrant compared to the opposing (*p* = 0.0479) and adjacent (*p* = 0.0150) quadrants. The *App*^+/+^ rpAD mice showed significant preference to the target quadrant compared to the adjacent quadrants (*p* < 0.05), but not the opposing quadrant. The *App*^+/+^C mice showed no significant preference for the target quadrant (Figure 3Bi). Furthermore, no significant effect of seed [*F*_(1, 47)_ = 0.0283, *p* = 0.867] or genotype [*F*_(2, 47)_ = 0.892, *p* = 0.417] was found on the cumulative distance from the target location.

When comparing the time spent in the target quadrant to chance performance, the *App*^−/−^ C [*t*_(14)_ = 2.144, *p* = 0.025], *App*^−/−^ rpAD [*t*_(14)_ = 3.191, *p* = 0.003], *App*^+/−^ rpAD [*t*_(2)_ =2.925, *p* = 0.0498], *App*^+/+^ C [*t*_(3)_ = 2.769, *p* = 0.035], and *App*^+/+^ rpAD [*t*_(8)_ = 2.732, *p* = 0.0129]-seeded mice spent significantly more time in the target quadrant compared to what would be predicted by chance. The *App*^+/−^ C mice did not show performance above chance [*t*_(6)_ = 1.244, *p* = 0.130]. Furthermore, when looking at whether the mice crossed the platform location during the probe trial, 83% of the *App*^−/−^ C mice, 92% of the *App*^−/−^ rpAD, 83% of the *App*^+/−^ C, 100% of the *App*^+/−^ rpAD, 50% of the *App*^+/+^ C, and 57% of the *App*^+/+^ rpAD mice showed at least one platform crossing. The swim pattern also shows that the *App*^−/−^ and *App*^+/+^ paths were much more directed in the target quadrant, whereas the *App*^+/−^ mice showed a much more diffuse pattern of swimming, with the path following the edge of the pool.

Lastly, no effect of sex was found on time in the target quadrant [*F*_(1, 41)_ = 0.345, *p* = 0.560].

Despite not all the mice showing preference for the target quadrant compared to the non-target quadrants, no significant effect of seed [*F*_(1, 47)_ = 0.0141, *p* = 0.906] or genotype [*F*_(2, 47)_ = 0.692, *p* = 0.506] was found on the time in the target quadrant.

To summarize, no significant effect of genotype or seed or overall significant differences were found in MWT performance between groups of the mice despite not all the groups showing significant learning or memory of the target location. The *App*^−/−^ mice showed learning and memory, the *App*^+/−^ mice did not, and only the *App*^+/+^ rpAD mice showed preference for the target quadrant despite the *App*^+/+^ C mice showing some evidence of learning, although all the groups showed significantly greater performance compared to chance, except for the *App*^+/−^ C mice.

The results presented show that 1 month following seeding both human tissue and Aβ protein increased Aβ plaque pathology, and this was dependent on the presence of the NL G F mutations on the *App* gene. When tested on the MWT, the mice showed they were able to learn and remember the location of the hidden platform except for the *App*^+/−^C mice. Despite the most extensive pathology, the *App*^+/+^ mice were able to successfully learn the task. However, the *App*^+/+^ mice showed the smallest proportion of the mice, showing at least one platform crossing, whereas the *App*^+/−^ mice showed relatively high occurrences of the mice crossing the platform at least once.

## Discussion

Here, we show that the initial pathology of AD—microglia activation and Aβ plaque deposition—is dependent on the combination of knocked-in genes and the seed. Each combination resulted in unique phenotypic expression of behavior, Aβ deposition, and the microglia activation pattern in young *App*^*NL*−*G*−*F*^ mice. One month following intracerebral seeding, prior to when the natural endogenous development of Aβ plaque deposition and microgliosis occurred, we found that Aβ plaque deposition and microgliosis increased in the *App*^+/+^ mice, but not in the *App*^−/−^ and *App*^+/−^ mice. It was not until 4 months following seeding did minimal Aβ plaque deposition occur in *App*^+/−^-seeded mice (Supplementary Figure 1). But no Aβ plaque pathology or microgliosis was found in the *App*^−/−^ mice (Supplementary Figure 1). The difference in Aβ deposition and microgliosis between the mice tested could be that the genes promote Aβ deposition or reduce the ability to slow Aβ deposition. Here we provide evidence suggesting the difference in pathology is due to a reduced ability of the App^+/+^ mice to slow Aβ deposition; likely due to the prion - like properties of Aβ and the role microglia play in the development and growth of Aβ plaque.

In the test of spatial learning and memory used, both the *App*^−/−^ and *App*^+/+^ mice showed evidence of learning the location of the hidden target. Due to only using 30-s trials, the learning curves were not as strong as those seen using 60-s trials, but the performance of the mice by the 6th day of the training was similar to tasks using longer trials; however, the increased days of training do result in better probe test performance (Mehla et al., 2019), suggesting that the task parameters resulted in similar patterns of learning compared to age matched, non-seeded controls, but, due to the reduced training volume before the testing, the no-platform probe trial becomes more difficult. However, no significant difference was found in the time spent in the target quadrant between the different groups in our study despite the *App*^+/−^ C mice, showing no preference for the target quadrant; this may be due to the small sample size, but the swim path suggests these mice did not learn the task. The impairment found in the *App*^+/−^ C mice is an effect that will have to be further investigated as well as an in-depth characterization of the *App*^+/−^ mice.

The *App* knock-in mice have humanized Aβ, but if one allele was producing murine Aβ, a disruption in the processing and function of Aβ during development may occur, specifically impairing the cerebrovascular system (Luna et al., 2013). This may not be occurring when only murine Aβ is present (*App*^−/−^) or when only human Aβ is present (*App*^+/+^). Unfortunately, we did not assess cerebrovascular health in these mice, but other work has shown that cerebrovascular dysfunction is a risk-factor AD (Esiri et al., 1999; Zhai et al., 2016). But understanding how the cerebrovascular system changes in the brain during AD progression is important for understanding AD pathobiology.

One facet of AD that is still not well-understood is the pathogenetic mechanisms leading to the difference in progression and phenotypic expression of AD (Schellenberg and Montine, 2012; Cohen et al., 2015). Our results and others' suggest that two general factors influence the difference in phenotypic expression: the type of Aβ and the underlying genetics. The underlying mechanism leading to these phenotypic differences may be the activation pattern of microglia in the brain.

Here, we showed that the relationship of microglia to Aβ plaque pathology was influenced by the type of seed and the presence of the three knock-in genes described. For example, in the *App*^+/+^ C mice, no significant correlation was found between the plaque count and the activated microglia, but, as the number of the microglia increased, there was a concomitant decrease in the average size of the plaque. In the *App*^+/+^-rpAD mice, as the plaque count increased so did the microglia; however, the plaque size did not correlate with the number of activated microglia. Despite the differences in correlation between the size of the plaque and the number of microglia cells, the overall number of microglia was not found to be significantly different between the control and the rpAD-seeded *App*^+/+^ mice.

The *App*^−/−^ mice did not develop plaque or microgliosis up to 7 months following seeding—or 9 months of age (not shown). The *App*^+/−^ mice did develop plaque 4 months following seeding but to a significantly lesser degree than the *App*^+/+^ mice at the same age (Supplementary Figure 1).

Despite finding that microglia and the plaque size and the count were significantly related, we were not able to directly discern the direction of this relationship. But, from the work of others, the elimination of microglia results in a reduction of Aβ plaque production early in the disease, but not in the later portions of the disease (Spangenberg et al., 2019; Saucken et al., 2020). Microglia are thought to internalize neuronally derived Aβ and begin the initial aggregating phase of Aβ before being deposited into the extracellular space (Spangenberg et al., 2019), suggesting that the activation of microglia may initially be causing the plaque deposition to protect the brain from the exogenously introduced agents, such as bacteria, viruses, such as the currently relevant SARS-COV2 virus, and exogenous Aβ (Eimer et al., 2018; Dominy et al., 2019; Montalvan et al., 2020; Wu et al., 2020); however, Aβ deposition could also occur due to an autoimmune response (Meier-Stephenson et al., 2022). We show that this plaque/microglia response is dependent specifically on the brain environment and the genetics underlying this phenotype as we found no microglia activation in the control or the rpAD-seeded *App*^−/−^ mice.

Microglia are known as the resident immune cells of the CNS and are known to interact with plaque to create a barrier. These innate immune cells (Webers et al., 2020) interact with plaque to control growth, development, and plaque morphology (Bolmont et al., 2008; Baik et al., 2016; Casali et al., 2020) and to prevent the toxic effects of Aβ_42_ (Condello et al., 2015). However, excessive uptake of Aβ can result in microglial death, resulting in the release of accumulated Aβ into the extracellular space and contributing to plaque growth (Baik et al., 2016). The activation of microglia cells with Aβ plaque pathology may explain why the *App*^+/+^ mice did not show impairment.

It is not uncommon for a peripheral inflammatory response to have effects on CNS function (Block, 2019). A poor microbiome, which is associated with inflammation, is associated with lower cognitive scores (Fröhlich et al., 2016; Gareau, 2016; Komanduri et al., 2021) and can also lead to AD (Thaiss et al., 2016; Shi et al., 2017; Lin et al., 2018). One potential mechanism that could explain why a poor microbiome is associated with lower cognitive could be due to disruption in tryptophan metabolism. Tryptophan metabolism is a key regulator of brain innate immunity (Meier-Stephenson et al., 2022) and a poor microbiota can lead to impaired tryptophan metabolism and serotonin signaling (Jenkins et al., 2016; Dinan and Cryan, 2017).

Further evidence that the etiology may be an immune response arises from fecal microbiota transplant experiments. The microbiota of the 6-month-old *App*^*NL*−*G*−*F*^ mice when transplanted into wild-type controls resulted in a disease phenotype; this effect was also sex and genotype specific (Kundu et al., 2022). The APP/PS1 mice transplanted with healthy fecal microbiota were found to have alleviated AD symptoms, such as a reduction in Aβ production and increased short chain fatty acid butyrate (Sun et al., 2019). Aged microbiota when transplanted was found to accelerate age and drive a pathological phenotype in young mice (Parker et al., 2022).

Lipid metabolism of microglia appears central to the functioning of microglia (Chausse et al., 2021), suggesting that disruption of these processes may impair microglia function and, therefore, their influence on plaque morphology and the innate immune system. As described above, the microbiota influences the immune system, and this appears to be through the maturation, function, and lipid metabolism of microglia in the CNS (Erny et al., 2015). Therefore, an impairment of the gut microbiome may be a central point in which AD begins. Its disruption leads to increased inflammation, disrupts microglia function and maturation, and, potentially, the metabolic processes of microglia, such as the processes related to managing Aβ plaque growth and toxicity (Bolmont et al., 2008; Condello et al., 2015; Baik et al., 2016).

Despite the described role of microglia in the etiology of AD, the role of Aβ oligomers cannot be overlooked. It is known that Aβ oligomers instead of fibrils and plaques are the most pathogenic Aβ species (Ashe, 2020). However, the toxicity of Aβ oligomers appears to be dependent on the temporal, spatial, and structural relationship to amyloid fibrils and dense core plaques (Liu et al., 2015). Liu et al. (2015) describe two classes of Aβ oligomers: Type 1 and Type 2. Type 2, while more abundant than Type 1, appears confined to the vicinity of plaque and does not impair cognition at levels relevant to AD. The Type-1 oligomers, however, are unrelated to amyloid fibrils and may, therefore, have a greater potential for neural dysfunction throughout the brain. It was concluded that, due to the containment of Type-2 oligomers to the Aβ plaques, the Type-2 oligomers are rendered functionally innocuous. Therefore, due to the intracerebral seeding of Aβ accelerating the nucleation and deposition of Aβ in the brain, a large portion of the Aβ oligomers in the brain may have been sequestered to the plaque, reducing the toxic effects of these oligomers, and preserving cognitive function. Yet microglia may be playing a role to contain the Type-2 Aβ oligomers inside the plaque, and it is not until later in the disease progression does the Type-2 Aβ oligomers leak out and begin damaging neurons throughout the brain (Liu et al., 2015). Unfortunately, we did not measure the levels of these two types of Aβ oligomers and, therefore, cannot determine if seeding altered the spatial or temporal characteristics of these oligomers. However, we do provide further evidence that Aβ monomers found in the seeds used can initiate Aβ aggregation in the *App*^*NL*−*G*−*F*^ mice.

We do acknowledge the limitations to this study. First, we focused only on spatial navigation learning and memory. In only testing spatial navigation, we were unable to make conclusions on the effects of seeding, or genotype in different cognitive domains. However, the MWT was originally designed to test the function of the HPC in memory, and one of the earliest impairments in AD is found in HPC memory. In future studies, additional behavioral tests should be included or a novel home cage-based assessment of rodent's behavior (Singh et al., 2019; Contreras et al., 2022) may offer insights into phenotypical expression of mouse genotypes and seeding and across time not found in traditional rodent behavioral testing. Furthermore, we did not measure soluble Aβ and, instead, focused on Aβ plaque load and characteristics throughout the brain and how this was associated with microglia. We, therefore, cannot make any conclusions on how seeding affected soluble Aβ. Lastly, while the control tissue had no rpAD Aβ, Aβ from the HPC where the tissue was collected could have induced the seeding response, which could explain the lack of difference in Aβ pathology 1 month following the seeding.

Given the recent failures of Aβ-targeted therapies to treat AD (Kurkinen, 2021), it is clear that the etiology of AD is not understood. The role of the immune system in the etiology of AD has been gaining interest (Jevtic et al., 2017). Recent hypotheses put forth have described that the initial Aβ plaque deposition that ultimately leads to AD occurs as a means to protect the brain from infection (Kumar et al., 2016; Eimer et al., 2018; Moir et al., 2018). While still speculative, our results along with others', suggest that the underlying genetic factors that contribute to AD may be closely related to the innate immune response and, specifically, the role of microglia. This immune response is determined by genetic and potentially epigenetic predisposition to the development of Aβ_42_ and the ratio of Aβ_42_ and Aβ_40_ but also the type of Aβ present in the brain, whether endogenous (Cohen et al., 2015, 2016) or exogenously introduced. It is well-known that both genetic and environmental factors influence the etiology of AD (McDonald et al., 2010), but, specifically, factors involving the immune system may be a novel approach to treat AD and age-related cognitive decline. Future studies should focus on microglia metabolism in health and disease and how this is influenced by the microbiome and the immune system.

## Data availability statement

The original contributions presented in the study are included in the article/Supplementary material, further inquiries can be directed to the corresponding author/s.

## Ethics statement

The animal study was reviewed and approved by Animal Ethics Committee of the University of Lethbridge.

## Author contributions

RS, MM, JS, DW, and SL: conceptualization. RS, SL, and DW: methodology. SL: software, validation, formal analysis, investigation, data curation, writing—original draft preparation, and visualization. RS, MM, DW, and JS: resources. SL, RS, MM, and DW: writing—review and editing and funding acquisition. RS and MM: supervision. SL and RS: project administration. All the authors have read and agreed to the published version of the manuscript.

## Funding

This research was funded by a CIHR grant awarded to RS (452424).

## Conflict of interest

The authors declare that the research was conducted in the absence of any commercial or financial relationships that could be construed as a potential conflict of interest.

## Publisher's note

All claims expressed in this article are solely those of the authors and do not necessarily represent those of their affiliated organizations, or those of the publisher, the editors and the reviewers. Any product that may be evaluated in this article, or claim that may be made by its manufacturer, is not guaranteed or endorsed by the publisher.
