# Supplementary material for: The effect of Aβ seeding is dependent on the presence of knock-in genes in the AppNL−G−F mice
Source: Front Dement. 2022 Sep 12;1:941879. doi: 10.3389/frdem.2022.941879 (PMC11285652; doi:10.3389/frdem.2022.941879)
Supplement: Supplementary file 1 [file Image_1.pdf]

Supplementary Material

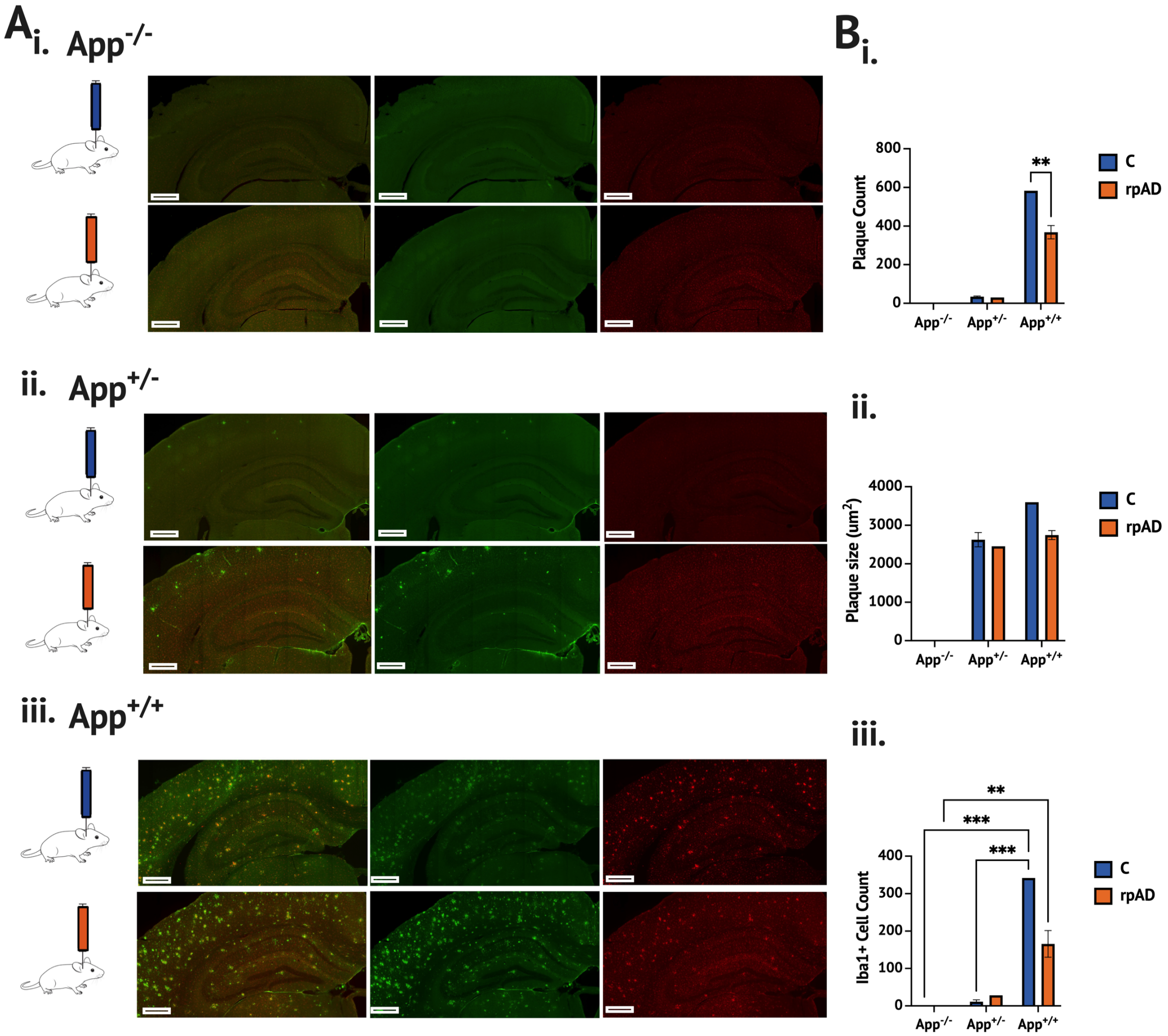

Supplementary Fig 1. Four months post seeding Aβ and microgliosis analysis. (**A**) representative photomicrographs Aβ (green) and microgliosis (red) pathology of (**i.**) *App*$^{-/-}$, (**ii.**) *App*$^{+/-}$, (**iii.**) *App*$^{+/+}$ mice. (**B**.) Quantification of (**i.**) plaque count, **(ii.)** plaque size, and **(iii.)** Iba1+ count. At six months the *App*$^{-/-}$ mice still showed no plaque or microgliosis (**Ai**) but the *App*$^{+/-}$ mice began showing plaque deposition and microgliosis with plaque size being comparable to the *App*$^{+/+}$ mice.

The mice in this experiment were allowed to age until six months of age (four months following seeding, when Aβ deposition naturally occurs in this model (Supplementary Figure 1). We found significant differences in plaque count between genotypes [$F(2, 10) = 110.0$, $p < 0.0001$]. The *App*$^{-/-}$ mice developed no plaque but the *App*$^{+/-}$ mice were found to have minimal plaque deposition but which was significantly greater than the *App*$^{-/-}$ mice. The *App*$^{+/+}$ mice still had significantly more plaque deposition than both the *App*$^{-/-}$ and *App*$^{+/+}$ mice ($p < 0.0001$). A significant effect of seed was found on plaque count [$F(1, 10) = 6.209$, $p = 0.0319$] as well as a significant gene x seed interaction [$F(2, 10) = 5.76$, $p = 0.022$]. A Sidak's multiple comparison revealed that the *App*$^{+/+}$ rpAD seeded mice had significantly greater plaque deposition compared to the *App*$^{+/+}$ C mice ($p = 0.0076$); however, the effect of seeding at this later time point will have to be investigated further. The genotype of the mice was also found to have a significant effect on plaque size [$F(2, 10) = 270.7$, $p < 0.0001$], as well as seed [$F(1, 10) = 6.871$, $p = 0.0255$]. A significant genotype x seed interaction was found [$F(2, 10) = 4.180$, $p = 0.048$].

Iba1+ cells were counted, and a significant effect of genotype was found [$F(2, 10) = 29.14$, $p < 0.0001$] but no effect of seed [$F(1, 10) = 3.084$, $p = 0.110$] or interaction was found [$F(2, 10) = 3.940$, $p = 0.055$]. The *App*$^{+/+}$ mice regardless of seed had significantly more Iba1+ cells compared to both the *App*$^{-/-}$ and *App*$^{+/-}$ mice.

Despite the effects of seed and genotype at this time point, the sample size may be too small to definitively state any conclusions, but future work should further assess behavioural and biochemical changes following seeding.
